# Targeting CCL20 inhibits subarachnoid hemorrhage-related neuroinflammation in mice

**DOI:** 10.18632/aging.103548

**Published:** 2020-06-21

**Authors:** Li-Shang Liao, Ming-Wei Zhang, Ying-Jiang Gu, Xiao-Chuan Sun

**Affiliations:** 1 Department of Neurosurgery, The First Affiliated Hospital of Chongqing Medical University, Chongqing 400016, China; 2 Department of Neurosurgery, The Affiliated Traditional Chinese Medicine Hospital of Southwest Medical University, Luzhou 646000, China

**Keywords:** CCL20, subarachnoid hemorrhage, neuroinflammation, CCR6, apoptosis

## Abstract

Recent evidence suggests that CC chemokine ligand 20 (CCL20) is upregulated after subarachnoid hemorrhage (SAH). Here, we investigated the functions of CCL20 in SAH injury and its underlying mechanisms of action. We found that CCL20 is upregulated in an SAH mouse model and in cultured primary microglia and neurons. CCL20-neutralizing antibody alleviated SAH-induced neurological deficits, decreased brain water content and neuronal apoptosis, and repressed microglial activation. We observed increased levels of CCL20, CC chemokine receptor 6 (CCR6), interleukin 1 beta (IL-1β), and tumor necrosis factor alpha (TNF-α), as well as of microglial activation in microglia treated with oxyhemoglobin (OxyHb). CCL20 or CCR6 knockdown reversed the effects of OxyHb on microglia. Conditioned medium from OxyHb-treated microglia induced neuronal apoptosis, while the percentage of apoptotic neurons in the conditioned medium from microglia transfected with CCL20 siRNA or CCR6 siRNA was decreased. We observed no decrease in OxyHb-induced apoptosis in CCL20-knockdown neurons. Conditioned medium from OxyHb-treated neurons led to microglial activation and induced CCR6, IL-1β and TNF-α expression, while CCL20 knockdown in neurons or CCR6 knockdown in microglia reversed those effects. Our results thus suggest CCL20 may be targeted to elicit therapeutic benefits after SAH injury.

## INTRODUCTION

Subarachnoid hemorrhage (SAH) is a very common clinical cerebrovascular syndrome characterized by acute onset and high lethality and disability rates that account for approximately 5%-15% of all stroke cases [1]. Recent studies have found that early brain injury (EBI) after SAH is the leading cause of death in patients [2]. Additionally, EBI triggers inflammation, which ultimately results in blood-brain barrier (BBB) disruption and neuronal degeneration [3]. Understanding the mechanisms underlying neuroinflammation could aid the discovery of new therapeutic targets against EBI after SAH as well as the development of better treatment strategies.

CC chemokine ligand 20 (CCL20), also known as liver and activation-regulated chemokine (LARC), Exodus-1, and macrophage inflammatory protein-3α (MIP-3α), belongs to the subfamily of small CC cytokine [4, 5]. Under normal and homeostatic conditions, CCL20 is expressed by a variety of epithelial [6, 7] and immune [5, 8] cells. CCL20 is normally expressed at a low basal level and is upregulated under various inflammatory conditions [9, 10]. Its upregulation contributes to the inflammatory cascade reaction. For example, hypoxia induced monocyte-derived CCL20 secretion in response to lipopolysaccharide (LPS), thereby promoting the recruitment of Th17 cells and osteoclastogenesis, which subsequently contributed to rheumatoid arthritis pathogenesis [10]. Additionally, CCL20 expression was increased in response to neuron-derived extracellular α-synuclein and contributed to α-synuclein-induced neuroinflammation in neurodegenerative diseases [11]. In spinal cord injury, CCL20-neutralizing antibody helps to recover motor functions and can reduce inflammation by inhibiting interleukin 1 beta (IL-1β), tumor necrosis factor alpha (TNF-α), and interleukin 1 (IL-6) [12]. The expression of CCL20 and its cognate receptor CC chemokine receptor 6 (CCR6) was upregulated in ischemic brain injury, while CCL20-neutralizing antibody reduced the volume of cerebral infarction [13]. Interestingly, a recent study reported that CCL20 expression is upregulated after SAH [14]. However, the contributions of CCL20 to the pathology of EBI after SAH and its underlying mechanisms are still unclear.

Here, we showed that CCL20 was upregulated in neurons and microglia after SAH and induced inflammatory reactions and neuronal apoptosis by increasing CCR6 activity. Therefore, our results suggest that targeting the CCL20/CCR6 pathway may represent a potential therapeutic strategy for SAH treatment.

## RESULTS

### CCL20 expression was increased after SAH

**Figure 1:**
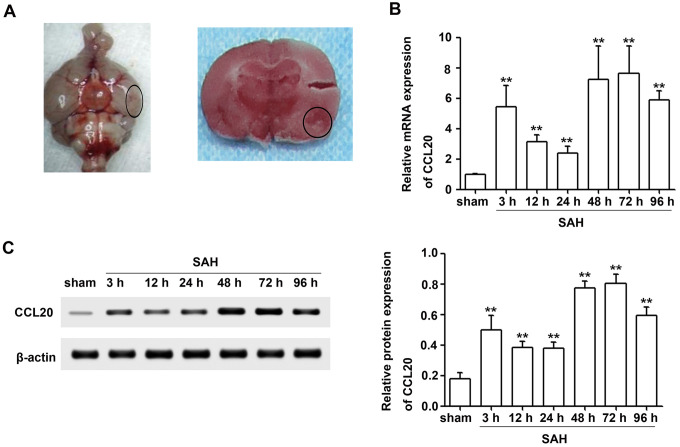
**CCL20 expression was increased after SAH.** (**A**) Representative picture of brains from the SAH groups and a schematic indicating the optimal brain region for further analyses (**B**) The CCL20 mRNA expression levels were determined by RT-qPCR (n = 5 per group). (**C**) The CCL20 protein expression levels were determined by Western blotting (n = 5 per group). Data were presented as mean ± SD. **P < 0.01 *vs*. the sham group.

To quantify the expression of CCL20 mRNA and protein in brain tissues after SAH, we created SAH models (Figure 1A). Brain tissues were collected from the right temporal cerebral for RT-qPCR and Western blotting analyses (Figure 1A). CCL20 mRNA expression was increased at all time points after SAH compared with the sham group and reached a peak at 72 h (Figure 1B). As shown in Figure 1C, CCL20 protein expression was also higher in the SAH group than in the sham group, which was consistent with the RT-qPCR results.

### CCL20 was mainly located in neurons and microglia after SAH

**Figure 2:**
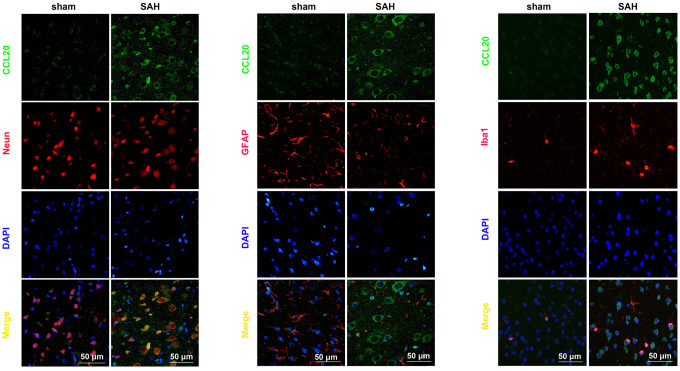
**CCL20 was mainly localized in neurons and microglia.** Double immunofluorescence staining was performed on mouse brain sections incubated with CCL20 (green) and NeuN (red), GFAP (red), or Iba1 (red). Cell nuclei were stained with DAPI (blue). Scale bar = 50 μm.

To elucidate whether a specific cell type was the main contributor to the increase in CCL20 expression after SAH, we performed double immunofluorescence staining to determine the localization of the immunofluorescence signals for CCL20 with NeuN (neuronal marker), GFAP (astrocytic marker), or Iba1 (microglial marker), respectively. As shown in Figure 2, increased CCL20 expression was mainly localized in neurons and microglia, but not in astrocytes at 24 h after SAH.

### CCL20-neutralizing antibody ameliorated neurological functions and brain edema after SAH

**Figure 3:**
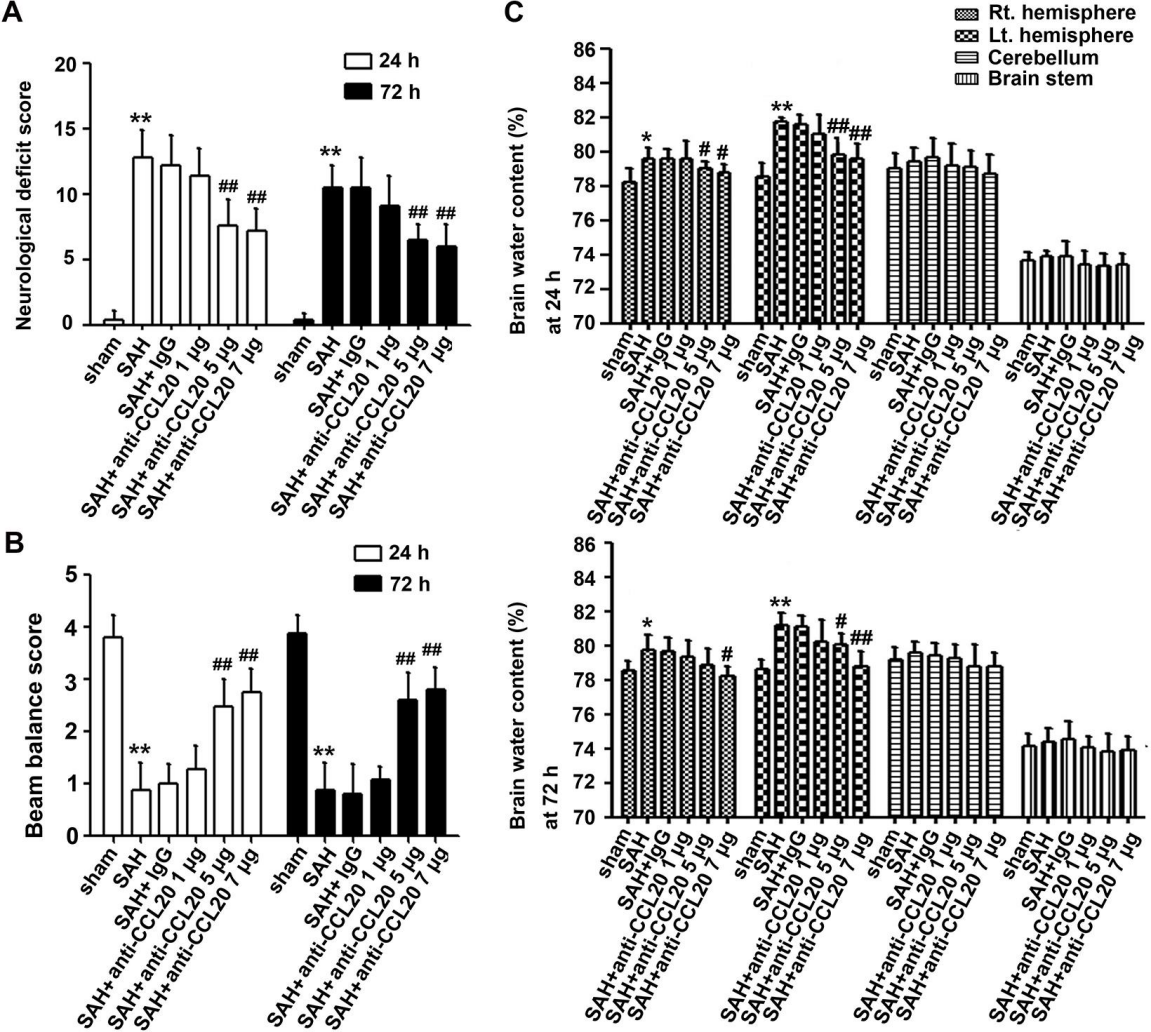
**The effects of CCL20 on neurological deficits and brain edema after SAH.** Various doses of CCL20-neutralizing antibody (1 μg/4 μl, 5 μg/4 μl and 7 μg/4 μl) or an equal volume IgG were administered into the brains of mice before SAH. Neurological deficits (**A**) (n = 15 per group), beam balance tests (**B**) (n = 15 per group) and brain edema (**C**) (n = 5 per group) were determined at 24 h and 72 h after SAH. Data were presented as mean ± SD. *P < 0.05, **P < 0.01 *vs*. the sham group. ^#^*P* < 0.01, ^##^*P* < 0.01 *vs*. the SAH+IgG group.

To assess the effect of CCL20 on neurological behavior at 24 h and 72 h after SAH, we performed neurological deficit analysis. As shown in Figure 3A, SAH induced neurological deficit at 24 h and 72 h. CCL20-neutralizing antibody treatment at a dosage of 1 μg/4 μl did not improve neurological deficits compared with the IgG treatment (Figure 3A). CCL20-neutralizing antibody treatment decreased the neurological deficit scores at the dosage of 5 μg/4 μl and 7 μg/4 μl compared with IgG treatment, but the difference in the effects between the two dosages were not significant (Figure 3A). As shown in Figure 3B, the beam balance scores were lower in SAH group than those in the sham group. CCL20-neutralizing antibody treatment at a dosage of 1 μg/4 μl did not improve the beam balance scores compared with the IgG treatment (Figure 3B). CCL20-neutralizing antibody treatment decreased the beam balance scores at 5 μg/4 μl and 7 μg/4 μl doses compared with the IgG treatment, but the difference in the effects between the two dosages were not significant (Figure 3B). Moreover, brain water content was higher in the SAH group than in the sham group (Figure 3C). There was no difference among the SAH group and the SAH+IgG group (Figure 3C). However, compared with the IgG treatment, CCL20-neutralizing antibody treatment at 5 μg/4 μl and 7 μg/4 μl doses dramatically decreased the brain water content (Figure 3C).

### CCL20-neutralizing antibody inhibited neuronal apoptosis and microglial activation after SAH

**Figure 4:**
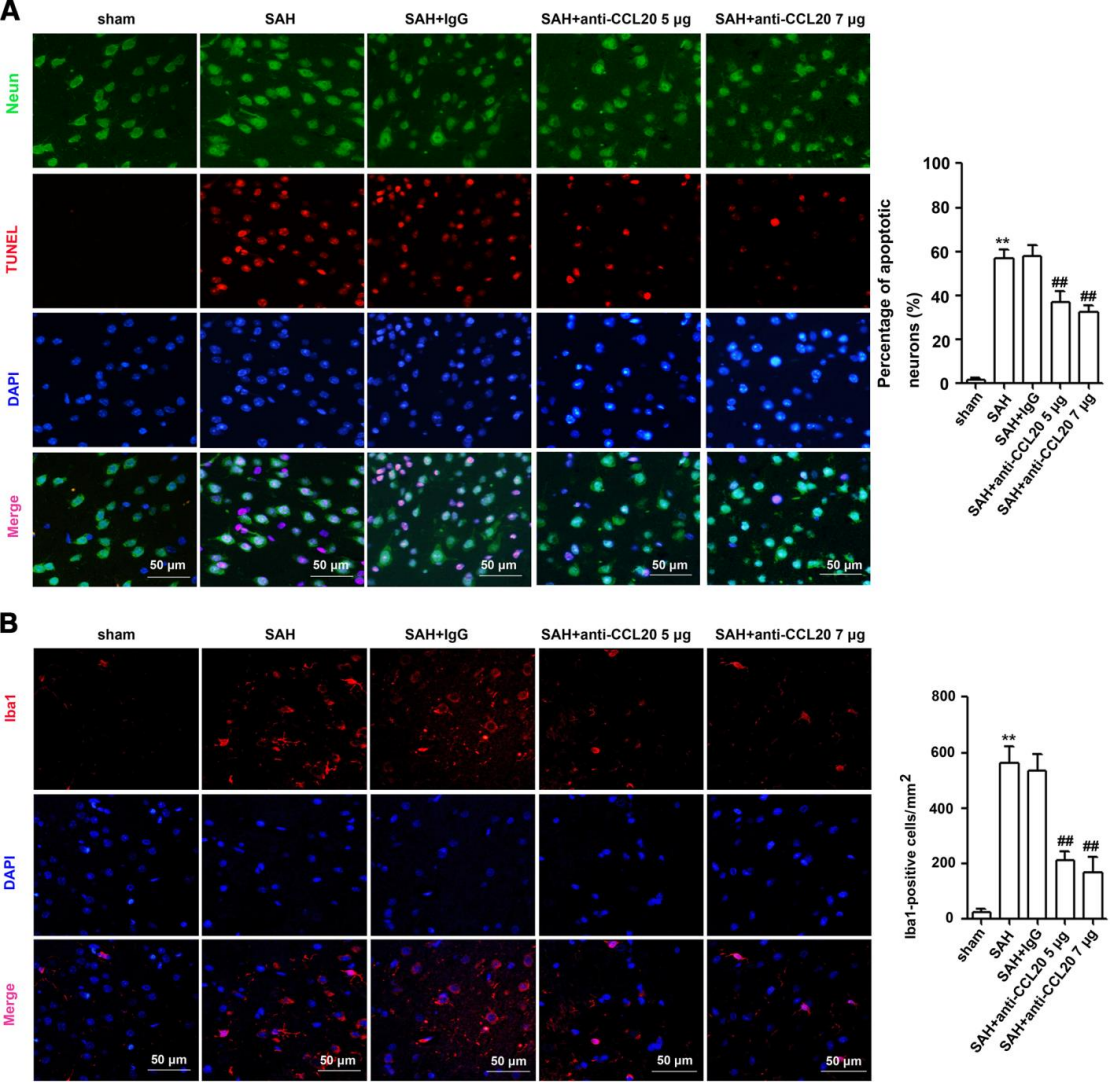
**The effects of CCL20 on the neuronal apoptosis and microglial activation after SAH.** (**A**) Neuronal apoptosis was determined by coimmunofluorescence staining for TUNEL (red), NeuN (green) and DAPI (blue) in the right temporal cerebral cortex at 24 h after SAH (n = 5 per group). Scale bar = 50 μm. (**B**) Microglial activation was detected through coimmunofluorescence staining for Iba1 (red) and DAPI (blue) in the cerebral cortical at 24 h after SAH (n = 5 per group). Scale bar = 50 μm. Data were presented as mean ± SD. **P < 0.01 *vs*. the sham group. ^##^*P* < 0.01 *vs*. the SAH+IgG group.

Since CCL20 colocalized with neurons and microglia, as illustrated above, we determined the effects of CCL20 on neuronal apoptosis and microglial activation by immunostaining. As shown in Figure 4A, the percentage of apoptotic neurons was increased in the SAH group compared with the sham group, which was reduced by CCL20-neutralizing antibody treatment at doses of 5 μg/4 μl and 7 μg/4 μl. As shown in Figure 4B, the number of Iba1-positive cells was increased in the SAH group compared with the sham group, which was reduced by CCL20-neutralizing antibody treatment at doses of 5 μg/4 μl and 7 μg/4 μl. Thus, our results suggest that increased CCL20 promotes EBI after SAH.

### CCL20 or CCR6 knockdown in microglia repressed microglial activation and microglia-mediated neuronal apoptosis *in vitro*

**Figure 5:**
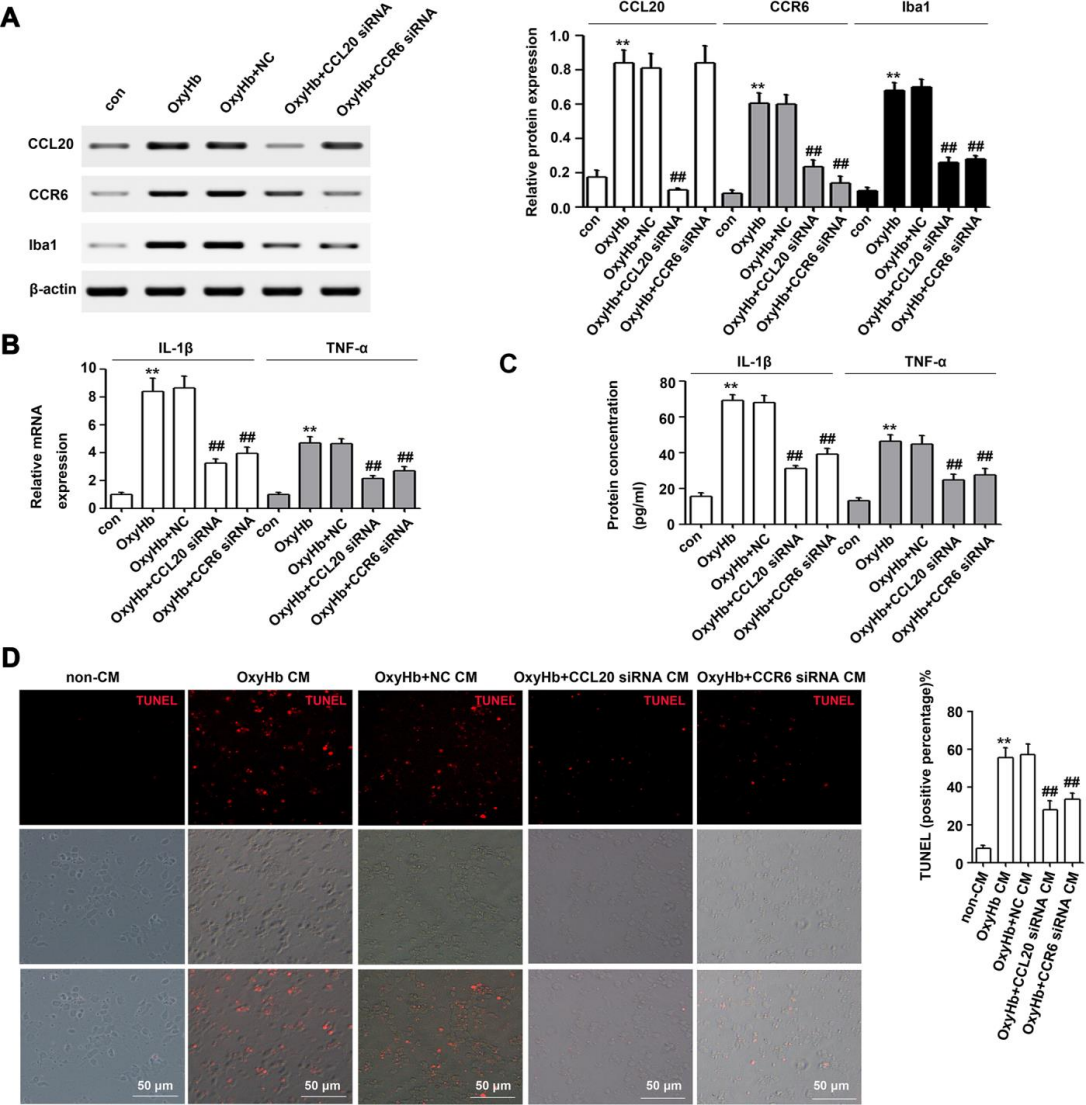
**The CCL20/CCR6 axis in microglia is involved in microglial activation and microglia-mediated neuronal apoptosis *in vitro*.** (**A**) Cultured microglia were transfected with the negative control siRNA (NC), CCL20 siRNA or CCR6 siRNA. After incubation for 72 h, microglia were exposed to OxyHb for 24 h. Next, Western blotting was used to measure the levels of CCL20, CCR6 and Iba1 (a microglial activation marker) (n = 3 per group). (**B**, **C**) The effects of CCL20 siRNA and CCR6 siRNA on OxyHb-induced IL-1β and TNF-α expression in microglia were analyzed by RT-qPCR (**B**) and ELISA (**C**) (n = 3 per group). (**D**) Cultured neurons were subjected to OxyHb-untreated conditioned medium (non-CM), OxyHb-treated conditioned medium (OxyHb CM), OxyHb CM from microglia pretreated with NC for 72 h (OxyHb+NC CM), OxyHb CM from microglia pretreated with CCL20 siRNA for 72 h (OxyHb+CCL20 siRNA CM), and OxyHb CM from microglia pretreated with CCR6 siRNA for 72 h (OxyHb+CCR6 siRNA CM) for 24 h. The apoptotic neurons were stained with TUNEL (red) (n = 3 per group). Scale bar = 50 μm. Data were presented as means ± SD. **P < 0.01 *vs*. the control group (con) or the non-CM group. Data were presented as mean ± SD. ^##^*P* < 0.01 *vs*. the OxyHb+NC group or the OxyHb+NC CM group.

Previous reports have shown that CCL20 promotes inflammation via its cognate receptor CCR6 [9]. Thus, we used western blotting to measure the levels of CCL20 and CCR6 protein in microglia after incubation with oxyhemoglobin (OxyHb) for 24 h. As shown in Figure 5A, both CCL20 and CCR6 protein expressions were upregulated in microglia after incubation with OxyHb compared with the control group. Additionally, Iba1 expression was higher in microglia after incubation with OxyHb than that in the control group (Figure 5A). Thus, we hypothesized that the CCL20/CCR6 axis may be involved in OxyHb-induced microglial activation. Subsequently, CCL20 and CCR6 expression in microglia was inhibited by siRNA. Western blotting results showed that CCL20 knockdown by siRNA reversed the OxyHb-induced CCL20 and CCR6 expression in microglia (Figure 5A). Additionally, OxyHb-induced Iba1 expression was repressed in microglia pretreated with CCL20 siRNA or CCR6 siRNA (Figure 5A). These results suggested that CCL20 induced microglial activation by promoting CCR6 activity in EBI after SAH. Moreover, we found increased IL-1β and TNF-α mRNA and protein levels in microglia treated with OxyHb, but CCL20 or CCR6 knockdown reversed the effects of OxyHb on microglia (Figure 5B, 5C). A previous study revealed that proinflammatory cytokines (IL-1β, IL-6 and TNF-α) from activated microglia caused neuronal cell death [15]. As shown in Figure 5D, conditioned medium from OxyHb-treated microglia induced neuronal apoptosis, while the percentage of apoptotic neurons in the conditioned medium of microglia transfected with CCL20 siRNA or CCR6 siRNA was decreased.

### CCL20 from neurons induced microglial activation by mediating CCR6 *in vitro*

**Figure 6:**
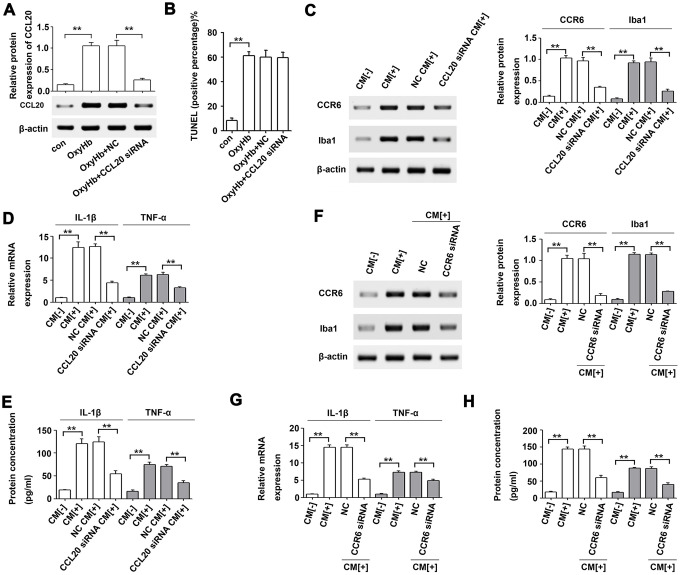
**Microglial activation was induced by neuron-derived CCL20 *in vitro*.** (**A**) The effect of CCL20 siRNA on OxyHb-induced CCL20 expression was analyzed by Western blotting (n = 3 per group). (**B**) The effect of CCL20 on OxyHb-induced neuronal apoptosis was analyzed by TUNEL assay (n = 3 per group). (**C**–**E**) Cultured microglia were subjected to OxyHb-untreated conditioned medium (CM[-]), OxyHb-treated conditioned medium (CM[+]), and OxyHb CM from neurons pretreated with CCL20 siRNA for 72 h (CCL20 siRNA CM[+]) for 24 h. Western blotting (**C**) was used to determine CCR6 and Iba1 levels (n = 3). The IL-1β and TNF-α expression levels were analyzed by RT-qPCR (**D**) and ELISA (**E**) (n = 3 per group). **P<0.01. (**F**–**H**) Cultured microglia pretreated with NC and CCR6 siRNA for 72 h were exposed to CM[+]. Then, Western blotting (**F**) was used to determine CCR6 and Iba1 levels (n = 3). RT-qPCR (**G**) and ELISA (**H**) were used to determine IL-1β and TNF-α levels (n = 3 per group). Data were presented as means ± SD. **P < 0.01.

**Figure 7:**
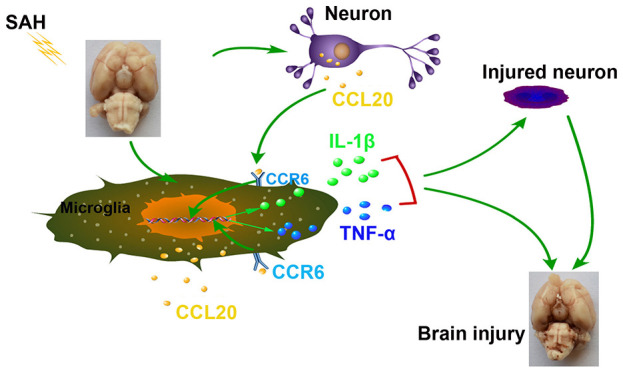
**Schematic summary of the role and mechanism of the CCL20/CCR6 axis in EBI after SAH.** The CCL20/CCR6 axis induced microglial activation and pro-inflammatory mediator release, thereby increasing neuronal apoptosis in SAH.

To further test the effect of CCL20 on neuronal apoptosis, primary cortical neurons were subjected to OxyHb treatment. As shown in Figure 6A, CCL20 protein expression was increased in OxyHb-treated cells compared with that in the control group, while CCL20 siRNA reduced the OxyHb-induced increase in CCL20 protein expression. However, CCL20 knockdown in OxyHb-treated neurons did not repress the neuronal apoptosis induced by OxyHb (Figure 6B). A previous study indicated that CCL20 from astrocytes stimulated IL-1β and inducible nitric oxide synthase expression in rat microglia [13]. Therefore, neuron-derived CCL20 may be involved in SAH-induced microglial activation. As shown in Figure 6C–6E, conditioned medium from OxyHb-treated neurons induced CCR6, Iba1, IL-1β and TNF-α in microglia, while CCL20 knockdown in neurons reversed the effects of conditioned medium from OxyHb-treated neurons on microglia. In addition, CCR6 knockdown in microglia also reversed the effects of conditioned medium from OxyHb-treated neurons on microglial activation and on IL-1β and TNF-α mRNA and protein levels (Figure 6F–6H). These results suggest that neuron-derived CCL20 induced microglial activation by mediating CCR6, thereby leading to neuronal apoptosis. Taken together, our results suggest that CCL20 may promote neuroinflammation by mediating CCR6 activation in EBI after SAH (Figure 7).

## DISCUSSION

The upregulation of CCL20, a member of the subfamily of small CC cytokines, contributes to the inflammatory cascade reaction and the development of disease pathogenesis [9–13, 16]. Moreover, CCL20 was increased after SAH [14]. In the present study, we observed that the levels of CCL20 mRNA and protein were increased after SAH (Figure 1B, 1C). Interestingly, we found that upregulated CCL20 was mainly localized in neurons and microglia, while little CCL20 was found in astrocytes (Figure 2). This suggested that SAH-induced CCL20 upregulation might promote EBI after SAH. To test this, we used a CCL20-neutralizing antibody to inhibit the activation of CCL20. As expected, CCL20-neutralizing antibody ameliorated the neurological functions and brain water content in EBI after SAH while inhibiting neuronal apoptosis and microglial activation (Figures 3, 4). Nevertheless, the underlying molecular mechanisms through which CCL20 regulates inflammation and neuronal damage in EBI after SAH require further investigation.

Previous reports have shown that CCL20 promotes inflammation via its cognate receptor CCR6 [9]. Thus, we hypothesized that CCL20 may induce inflammation in microglia by activating CCR6 in EBI after SAH. Our results confirmed this hypothesis. We found that CCL20 knockdown reversed OxyHb-induced CCR6 expression in microglia (Figure 5A). Moreover, CCL20 siRNA or CCR6 siRNA reversed OxyHb-induced microglial activation and the expression of the pro-inflammatory factors IL-1β and TNF-α (Figure 5B, 5C). Accumulated evidence has shown that pro-inflammatory and cytotoxic mediators such as IL-1β and TNF-α secreted by activated microglia after SAH contribute to neuronal dysfunction and cell death [17, 18]. Our results showed that CCL20 siRNA or CCR6 siRNA reversed neuronal apoptosis induced by conditioned medium from activated microglia (Figure 5D). Therefore, the CCL20/CCR6 axis contributed to microglial activation-induced neuronal apoptosis in EBI after SAH. Moreover, we found that CCL20 was localized in neurons and was upregulated after SAH (Figure 2). Based on recent reports and our above results, we hypothesized that CCL20 up-regulation induces neuronal apoptosis in SAH. However, our studies first showed that CCL20 knockdown in OxyHb-treated neurons did not affect the neuronal apoptosis induced by OxyHb (Figure 6B). Thus, these results suggested that the effect of CCL20 on neuronal apoptosis after SAH may be due to the microglial activation induced by CCL20. Moreover, one previous study indicated that CCL20 from astrocytes stimulated IL-1β and inducible nitric oxide synthase expression in rat microglia [14]. Therefore, CCL20 from neurons may be involved in SAH-induced microglial activation and pro-inflammatory mediator release. As shown in Figure 6C–6H, increased microglial activation and IL-1β and TNF-α expression levels were observed in microglia exposed to conditioned medium from OxyHb-treated neurons, while CCL20 knockdown in neurons or CCR6 knockdown in microglia reversed the effects of conditioned medium from OxyHb-treated neurons. These results suggest that CCL20 from neurons induced microglial activation by activating CCR6, thereby leading to neuronal apoptosis.

In conclusion, our results demonstrated that activation of the CCL20/CCR6 axis in EBI after SAH induced microglial activation and pro-inflammatory mediator release, thereby increasing neuronal apoptosis. Although additional research is required to understand the mechanisms underlying SAH, our results suggest that CCL20 may be a promising therapeutic agent that could be targeted to treat SAH.

## MATERIALS AND METHODS

### Animals

Adult male C57BL/6J mice (10-12 weeks old) were purchased from Southwest Medical University (Luzhou, China), and housed at a constant temperature (22 ± 1°C) with a 12:12 dark/light cycle. Food and water were available *ad libitum* for mice.

### Mouse model of SAH

The mouse model of SAH was established as previously reported [19, 20]. Briefly, mice were anesthetized with xylazine (10 mg/kg) and ketamine (12 mg/kg) and maintained at 37.0 ± 0.5 °C during surgery. The right common carotid artery was exposed by a midline incision of the neck. The external carotid artery (ECA) was isolated and ligated. A blunted 5-0 monofilament nylon suture was advanced into the internal carotid artery (ICA). The suture was advanced distal to the right anterior cerebral artery (ACA)-middle cerebral artery (MCA) bifurcation until resistance was felt, and then advanced 3.0 mm further to perforate the right ACA. The suture was immediately withdrawn to induce SAH. Sham-operated mice underwent the same procedures except that the suture was withdrawn without puncturing an artery.

### *In vivo* CCL20-neutralizing antibody treatment in SAH mouse brains

*In vivo* CCL20-neutralizing antibody treatment was administered as described previously [21]. Three treatment doses were prepared at concentrations of 1 μg, 5 μg and 7 μg in 4 μl buffer containing 0.1 μmol/μl Tris-glycine, 0.15 μmol/μl NaCl, 0.05% sodium azide, and 30% glycerol. A total volume of 4 μl normal rabbit IgG or CCL20-neutralizing antibody were stereotaxically injected into the right lateral ventricle (Bregma: -2.2mm, dorsoventral: 3mm, lateral: 1mm) 1 h before SAH.

### Small interfering RNA (siRNA) and transfection

The CCL20 siRNA, CCR6 siRNA and negative control siRNA (NC) used in this study were designed and synthesized by GenePharma (Shanghai, China). The siRNA sequences are listed in Table 1. A total of 5 × 10^5^ microglia or neurons were plated in 6-well plates and transfected using Lipofectamine® 2000 Reagent (Thermo Fisher Scientific, Inc., Waltham, MA, USA) according to the manufacturer’s protocol.

**Table 1:** The sequences of siRNA and RT-qPCR primers.

**Name**	**Sequences**
CCL20 siRNA sense	5′- CCGAUGAAGCUUGUGACAUUAdTdT-3′
CCL20 siRNA antisense	5′-UAAUGUCACAAGCUUCAUCGGdTdT-3′
CCR6 siRNA sense	5′- GUGUAUGAGAAGGAAGAAUAAdTdT-3′
CCR6 siRNA antisense	5′-UUAUUCUUCCUUCUCAUACACdTdT-3′
NC sense	5′- UUCUCCGAACGUGUCACGUdTdT-3′
NC antisense	5′-ACGUGACACGUUCGGAGAAdTdT-3′
CCL20 Fwd	5′- CGACTGTTGCCTCTCGTACA-3′
CCL20 Rev	5′- AGGAGGTTCACAGCCCTTTT-3′
β-actin Fwd	5′-GGAGATTACTGCCCTGGCTCCTA-3′
β-actin Rev	5′- GACTCATCGTACTCCTGCTTGCTG-3′
TNF-α Fwd	5′- CCTGTGAGGAGGACGAACA-3′
TNF-α Rev	5′- TTGAGCCAGAAGAGGTTGAG-3′
IL-1β Fwd	5′- CATCAGCACCTCTCAAGCAG-3′
IL-1β Rev	5′- AGTCCACATTCAGCACAGGA-3′

### Primary cortical neuron culture and conditioned medium stimulation experimental design

Primary cortical neural cells were cultured as described previously [22]. Briefly, cortical tissues were isolated to prepare cell suspensions from 1-day-old neonatal mouse brains. After sectioning under sterile conditions, the cortical tissues were digested with 0.125% trypsin (Gibco, CA, USA) at 37 °C for 30 min and filtered using a 40-μm strainer (Millipore, Darmstadt, Germany) and centrifuged at 1500 r/min for 10 min. Then, the cells were resuspended using DMEM/F12 (Gibco) with 10% fetal bovine serum (FBS) (Gibco) and seeded into poly-D-lysine-coated 6-well plates at a density of 6×10^5^ cells per well. After 2 h, the culture medium was replaced with neurobasal medium (Gibco) containing 2% B27. After 72 h, half of the culture medium was replaced with fresh medium containing arabinosylcytosine (2.5 μg/ml) (Santa Cruz Biotechnology, CA, USA) every three days. Neurons were stimulated with 25 μmol/L OxyHb for 24 h to mimic SAH conditions as described previously [22]. After incubation with OxyHb, neurons were collected for further analysis.

Microglia-conditioned medium was prepared as described previously [22, 23]. Briefly, microglia were cultured in DMEM at a concentration of 25 μmol/L OxyHb at 37°C for 24 h. Then, the conditioned medium was collected and centrifuged at 10,000 r/min for 5 min. The neuronal medium was replaced with the Microglia-conditioned medium and incubated at 37 °C for 24 h, and neurons were collected for further analysis.

### Microglia culture and conditioned medium stimulation experimental design

Primary microglia were obtained from 1-day-old neonatal mouse brains as described previously [22, 24]. Briefly, cortical tissues were obtained and sectioned under sterile conditions. Cortical tissues were then digested with Hank’s balanced salt solution (Gibco) containing 0.05% trypsin. The cell suspension was filtered using a 70-μm filter and centrifuged at 1000 rpm for 5 min. Subsequently, the cells were plated onto poly-D-lysine-coated flasks. After 24 h, the culture medium was replaced with fresh DMEM/F12 (Gibco) with 20% FBS. The culture medium was refreshed every other day. After nine days, the flasks were shaken at 250 rpm for 2 h. The microglia were collected and seeded in 6-well plates. Microglia were stimulated with 25 μmol/L OxyHb for 24 h to mimic SAH condition. After incubation with OxyHb, microglia were collected for further analysis.

Similar to microglia-conditioned medium preparation, neuron-conditioned medium was obtained as follows: neurons were cultured in DMEM at a concentration of 25 μmol/L OxyHb at 37°C for 24 h. The conditioned medium was collected and centrifuged at 10,000 r/min for 5 min. The microglia medium was replaced with the neuron-conditioned medium and incubated at 37°C for 24 h, and microglia were collected for further analysis.

### Neurological deficit scores and SAH grade

Neurological deficit scores were evaluated at 24 h and 72 h after SAH in a blinded manner as previously reported [25]. Briefly, neurological deficit scores is graded via the scale of 0-18 (0 = normal function; 18 = maximal deficit). Thus, higher scores signified more severe neurological deficits. The mice after SAH were measured by the SAH grade score. The SAH score is evaluated to ensure that the model is worked. Briefly, the basal cistern was divided into six parts. Each part was blindly evaluated a grade from 0 to 3 based on the amount of the blood clot in subarachnoid space [26]. The total scores ranged from 0 to 18. Mild SAH mice with a score below 8 points were excluded because there were no significant difference neurological impairments between sham and both treated and untreated mild SAH groups [27].

### Beam balance test

Beam balance tests were performed to measure the ability of mice to walk on a narrow cylindrical wooden beam at 24 h and 72 h after SAH in a blinded manner. Each mouse was placed on the beam and allowed to walk freely for 60 s. Then, the score was evaluated as follows: 0, no walking and falling; 1, no walking, but remains on the beam; 2, walking but falling; 3, walking less than 20 cm; 4, walking beyond 20 cm [28].

### Brain water content

Mouse brains were quickly divided into four segments (left hemisphere, right hemisphere, cerebellum, and brain stem) at 24 h and 72 h after SAH. Every segment was weighed immediately to obtain the wet weight (WW) and then kept in an oven for 72 h at 100 °C to obtain the dry weight (DW). The percentage of brain water content = [(WW − DW)/WW] × 100% [29].

### Terminal deoxynucleotidyl transferase (TdT)-mediated dUTP nick end labeling (TUNEL) staining

A cell apoptosis detection kit (Yeasen, Shanghai, China) was used to detect apoptotic neurons, previously reported [21]. Briefly, the brain sections were deparaffinized in xylene and rehydrated through graded alcohol solutions. The sections were then incubated in Triton X-100 for 10 min. Next, tissue sections were double stained using the NeuN antibody (Abcam Biotechnology) and TUNEL kit. The cell nuclei of neurons were stained with DAPI, and the collocation of red (TUNEL), green (NeuN) and blue (DAPI) indicated the apoptotic neurons. Four continuous pictures of the right temporal cerebral cortex were analyzed under a fluorescence microscope (× 200), and the average number of TUNEL-positive cells and NeuN-positive neurons in the fields as cells/mm^2^ was calculated. The mean value was obtained from five mice. Data were presented as the percentage of apoptotic neurons (%).

In addition, we also measured the neuronal apoptosis *in vitro* using the TUNEL kit. We quantified the percentage of apoptotic neurons among the total amount of neurons. To this end, we computed the mean percentage of apoptotic neurons from three images (× 200 magnification) per sample, and three samples per group. The mean percentage was obtained from three images.

### Immunofluorescence

Immunofluorescence staining was performed as described previously [30, 31]. Briefly, the brain sections were prepared as above. The rehydrated sections were incubated in Triton X-100 for 30 min, and were blocked with 1% bovine serum albumin (BSA) for 1 h at 37°C. The sections were then incubated overnight at 4°C with the following primary antibodies: rabbit anti-mouse CCL20 antibody (Abcam Biotechnology, Cambridge, UK), mouse anti-mouse Iba1 antibody (Santa Cruz Biotechnology), mouse anti-mouse NeuN antibody (Abcam Biotechnology), and mouse anti-mouse GFAP antibody (Abcam Biotechnology). After washing with TBST, the sections were incubated with goat anti-rabbit/mouse secondary antibody (Thermo Fisher Scientific) at 37°C for 1 h. Cellular nuclei were stained with DAPI (Beyotime, Jiangsu, China) at 37°C for 10 min. Images were captured using a microscope with a digital camera (Olympus, Tokyo, Japan).

### Western blotting

Western blotting was performed as described previously [32]. Briefly, the cells or brain samples were lysed using RIPA lysis buffer (Beyotime). A BCA protein assay kit (Beyotime) was then used to determine the protein concentrations. The protein samples (30 μg per lane) were mixed with loading buffer and were analyzed by SDS-PAGE. The protein samples were transferred onto PVDF membranes (Millipore Corporation). The membranes were blocked in 5% BSA (1 h at room temperature) and were incubated overnight at 4°C with the following primary antibodies: rabbit anti-mouse CCL20 antibody (Abcam Biotechnology), rabbit anti-mouse CCR6 antibody (Abcam Biotechnology), mouse anti-mouse Iba1 antibody (Santa Cruz Biotechnology), and rabbit anti-mouse β-actin antibody (Abcam Biotechnology).

### Reverse transcription quantitative polymerase chain reaction (RT-qPCR)

Total RNA was isolated from tissues or cultured cells using TRIzol (Invitrogen, Grand Island, NY, USA). Reverse transcription was performed using M-MLV-RTase (Promega Corp., Madison, WI, USA) at 42°C for 1 h. The expression of CCL20, IL-1β and TNF-α was analyzed using a SYBR Premix Ex Taq kit (TaKaRa, Dalian, China). The relative gene expression was normalized to β-actin expression using the 2^-ΔΔCt^ analysis method. All primers are listed in Table 1.

### Enzyme-linked immunosorbent assay (ELISA)

Microglial culture medium was collected and centrifuged at 12,000 rpm for 20 min. The levels of IL-1β and TNF-α were determined with ELISA kits (Abcam Biotechnology) according to the manufacturer’s instructions.

### Statistical analysis

Data were analyzed by using IBM SPSS 21.0 software (IBM., Chicago, IL, USA) and are presented as the mean ± SD. Significant differences between groups were analyzed using Student’s *t* test or one-way ANOVA. *P* < 0.05 was considered statistically significant.

### Ethics statement

This study has been conducted in accordance with the ethical standards and according to the Declaration of Helsinki and according to national and international guidelines and has been approved by the Southern Medical University Ethics Committee.
